# Assessing Landscape Constraints on Species Abundance: Does the Neighborhood Limit Species Response to Local Habitat Conservation Programs?

**DOI:** 10.1371/journal.pone.0099339

**Published:** 2014-06-11

**Authors:** Christopher F. Jorgensen, Larkin A. Powell, Jeffery J. Lusk, Andrew A. Bishop, Joseph J. Fontaine

**Affiliations:** 1 School of Natural Resources, University of Nebraska–Lincoln, Lincoln, Nebraska, United States of America; 2 Nebraska Cooperative Fish and Wildlife Research Unit, University of Nebraska–Lincoln, Lincoln, Nebraska, United States of America; 3 Nebraska Game and Parks Commission, Lincoln, Nebraska, United States of America; 4 U.S. Fish and Wildlife Service, Rainwater Basin Joint Venture, Grand Island, Nebraska, United States of America; 5 U.S. Geological Survey, Nebraska Cooperative Fish and Wildlife Research Unit, University of Nebraska–Lincoln, Lincoln, Nebraska, United States of America; University of New South Wales, Australia

## Abstract

Landscapes in agricultural systems continue to undergo significant change, and the loss of biodiversity is an ever-increasing threat. Although habitat restoration is beneficial, management actions do not always result in the desired outcome. Managers must understand why management actions fail; yet, past studies have focused on assessing habitat attributes at a single spatial scale, and often fail to consider the importance of ecological mechanisms that act across spatial scales. We located survey sites across southern Nebraska, USA and conducted point counts to estimate Ring-necked Pheasant abundance, an economically important species to the region, while simultaneously quantifying landscape effects using a geographic information system. To identify suitable areas for allocating limited management resources, we assessed land cover relationships to our counts using a Bayesian binomial-Poisson hierarchical model to construct predictive Species Distribution Models of relative abundance. Our results indicated that landscape scale land cover variables severely constrained or, alternatively, facilitated the positive effects of local land management for Ring-necked Pheasants.

## Introduction

Habitat management and restoration are fundamental components of conservation science [1], [2], [3], [4], [5] and are routinely identified as the primary means to improve population viability for species of social-economic [6], [4], [7], [8] or conservation concern [9], [10], [11]. Although habitat management success is often measured by the ability to produce a particular suite of vegetative structure and composition, ultimately success must be gauged by the population responses of target faunal species. Unfortunately, despite our ability to routinely produce ‘suitable’ vegetative conditions, habitat management actions too often fail to meet the population expectations of managers e.g., [12], [13], [14], [15]. Understanding why populations fail to respond to apparently suitable habitat conditions represents a true conservation challenge which necessitates reconsidering the underlying mechanisms that drive species-habitat relationships.

Recognizing that individuals select among available habitats based on a set of environmental cues is fundamental to habitat selection theory, and therefore is useful in predicting habitat suitability [16], [17]. The utilization of conservation tools which translate ecological theory into spatial species-habitat relationships, such as Species Distribution Models (SDMs), is therefore an effective population management strategy [18], [19]. Although habitat preferences have evolved to predict habitat suitability, the spatial scale at which individuals select and use habitat varies based on life history and mobility [20], [21], [22]. Many studies have demonstrated the importance of site-level habitat attributes [23], [24], [25], yet recent research has increasingly acknowledged that communities and other biological interactions are influenced by ecological factors across multiple spatial scales [26], [27], [28], [29], [30], [31], [32], [33]. Ignoring the fact that ecological processes act across spatial scales [30] reduces the efficacy of habitat management and can drain limited financial and ecological resources, or worse, harm the species or community in consideration (*i.e.,* ecological trap) [34]. Furthermore, public perception may change in concert with the success or failure of a management action, potentially dictating the future direction of policy and governance [35], [36]. To improve management efficacy, management plans must be based on ecological mechanisms, many of which can be integrated in to SDMs [18]. In particular, we suggest that emphasis should be focused on ecological factors that constrain management success, especially those factors which operate at spatial scales relevant to the biology of the species or communities of interest. Therefore, associating land cover variables with species occurrence or abundance on a spatial scale relevant to the species, potentially through the use of an SDM, may provide insight into how individuals make habitat decisions, and consequently, what constitutes suitable habitat [21].

Effective conservation practices may be particularly important in highly altered systems, such as agro-ecosystems. Over the past 50 years, agro-ecosystems throughout Europe and North America have been increasingly exposed to land-use intensification and development, causing extensive losses in ecosystem functions and corresponding species declines [37], [38]. Farmland and grassland birds, for example, have declined significantly over the past half century [39], [40], and therefore are at the forefront of agro-ecosystem conservation. In North America, the Conservation Reserve Program (CRP) is one example of an agro-ecosystem conservation practice that is widely regarded to be beneficial to wildlife, including farmland birds [38], [41], [42], [43]. Yet, despite significant successes incorporating CRP into the landscape, managers too often witness less-than-desirable management outcomes [12], [14]. The dynamic nature associated with agriculturally dominated landscapes provides a perfect opportunity to explore species-habitat relationships and identify why farmland birds fail to respond to apparently suitable habitat improvements. To understand how farmland bird conservation efforts may be constrained, we must understand and address ecological interactions at both the land management level and in the surrounding landscape to ask the question: Are local habitat conservation programs constrained by the surrounding landscape configuration and composition? Our objective was to assess the relationships between land cover variables measured at two spatial scales, both of which are either relevant to the biology of the species or land management, and species abundance. We evaluated whether the composition and context of the landscape affects species response to local habitat conservation programs. In addition, we utilized species’ relationships to topography and land cover to develop a SDM, providing habitat managers a means to visualize species response to complex species-habitat interactions.

## Materials and Methods

### Study Species

Originally introduced to the United States in the early 1900’s [45], the Ring-necked Pheasant (*Phasianus colchicus*) prospered in the agro-ecosystems of the Midwest and Great Plains. Pheasant populations thrived in landscapes containing a diversity of crop types established over a variety of field sizes [46]. As pheasant populations grew, their importance as an upland game species increased throughout much of North America, providing hunters a substitute for declining native grouse species. However, despite being a generalist and relatively resilient to human disturbance, Ring-necked Pheasant populations have experienced dramatic declines over the past 50 years [40]. Given the social and economic value of Ring-necked Pheasants, the dramatic population decline has sparked intense research and conservation efforts from agencies and non-government organizations throughout the United States [46], [47], [48], [49], [50]. Still, despite considerable efforts to conserve Ring-necked Pheasant populations, often management activities have proven unsuccessful [44], [51], [52] and the landscape context may be critical to productivity [53].

### Data Collection and Preparation

**Figure 1:**
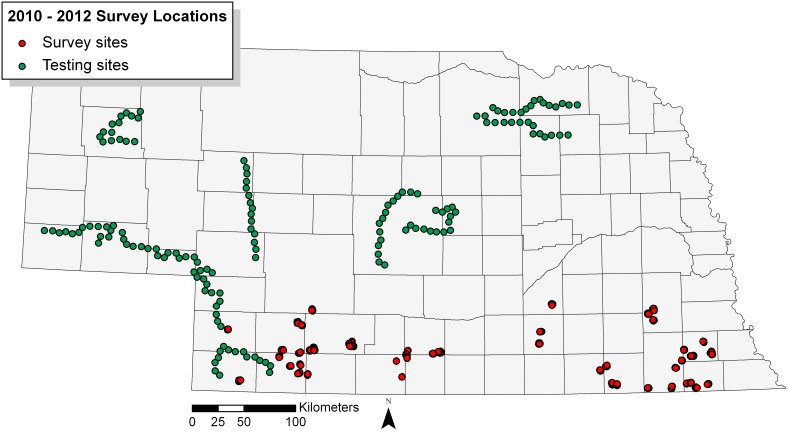
A map of Ring-necked Pheasant survey sites distributed throughout Nebraska. Ring-necked Pheasant abundance was recorded at 405 survey sites distributed throughout 45 state Wildlife Management Areas and private property enrolled in the Open Fields and Waters program located in southern Nebraska (red points). Survey data was used to fit statistical models, which were evaluated using an independent testing dataset consisting of 150 survey sites evenly distributed across 10 road-transects (green points).

During April through July of 2010–2012, we conducted aural surveys (2010, n = 648; 2011, n = 1161; and 2012, n = 1146) using a 500-m bounded distance-sampling method [54], [55] to estimate pheasant abundance at sites located throughout 17 counties in Nebraska (Figure 1). Approval by the University of Nebraska – Lincoln Institutional Animal Care and Use Committee (IACUC) was not necessary as no animals were directly handled or harmed in our surveys. Surveys began 15 minutes before sunrise and ended at 10∶00 a.m., when aural detection rates are most consistent across all species [56], and during which the maximum vocalization rate for Ring-necked Pheasants occurs [57]. All surveys were conducted on Nebraska Game and Parks Commission’s Wildlife Management Areas and private property enrolled in the Open Fields and Waters program. The Nebraska Game and Parks Commission’s Wildlife Division permitted the use of state lands and private lands open to public hunting. The field studies did not involve endangered or protected species and were conducted on various property locations across southern Nebraska (*see* List S1). Study sites had a minimum of a quarter-section (64 hectares) of contiguous grassland, the minimum habitat size assumed necessary to support viable Ring-necked Pheasant populations at a local spatial scale [53]. Although constraining the minimum habitat size ensures we are surveying suitable management areas for Ring-necked pheasants, it may also bias the modeling. This potential confounding effect on our modeling efforts caused by our site selection is reduced using a random survey design for establishing our survey points. We randomly selected nine survey points at each site using a minimum spacing of 300 meters and sampled each point three times each season, equally spacing time intervals between survey rounds. This random spacing of our survey points ensures there is equal potential for points bordering study sites to have less grassland in the surround area than points towards the center. We recorded every individual seen or heard during a 3-minute period and used a laser range finder to measure distance from observer to suspected location. Counting the number of male vocalizations and the number of individuals seen per a fixed period of time is widely held as an appropriate means of sampling Ring-necked Pheasants [58], [59], [60], [61], [62]. Inclement weather, including fog, drizzle, prolonged rain, and wind greater than 20 km/h resulted in ending the survey prematurely.

In order to test the predictive performance of our SDM resulting from our analysis, in 2012 we established 10 roadside transects outside of the original study area, each containing 15 survey locations, where each location was spaced roughly 5 km apart (Figure 1). Roadside transects allowed us to sample over large areas in a short amount of time, but may lead to potential biases based on our sampling design. For example, land cover types, such as the percent grassland within 1-km radius, may tend to be similar surrounding road ways and may not significantly differ between locations. In addition, the potential for edge effects to bias our abundance estimates increases by sampling strictly along roadways. Because it was unlikely home range would significantly change during the breeding season [50] and each transect was visited three times, we used the maximum number of individuals detected over the three visits for each survey location as the observed testing dataset. By using the maximum number of individuals detected, we assume population closure, where the same individuals present during the first survey continue to be present and available for counting for all repeated visits.

Land cover variables were derived from the Rainwater Basin Joint Venture Nebraska Landcover dataset with a 30×30-m resolution (unpublished data). The land cover dataset had a 70% success rate based on an accuracy assessment of 1,280 survey points sampled throughout much of the state. Generalized land cover classes had even a higher success rate (95% overall accuracy), yet the per class estimates of accuracy indicated that certain land cover classes were more reliable than others (unpublished data). Individual land cover types were generalized into six cover classes which we predicted *a priori* to influence Ring-necked Pheasant populations (Conservation Reserve Program grasses, grass, trees, small grains, row crops, and wetlands) and reclassified into six binary raster layers, where 1 is “presence” and 0 is “absence” of the cover type at a given location (e.g. trees). We wanted to assess both local (relevant to habitat management) and landscape effects (relevant to the species) on Ring-necked Pheasants, therefore we implemented the Circular Focal Statistics Tool in ArcGIS 10.0 (ESRI, Redlands, California) and calculated the proportion of habitat at both a land management scale (1 km radius), and a landscape scale (5 km radius). We selected a 1 km radius window (314 ha) to approximate one section (259 ha), a unit of land commonly used in an agriculturally dominant landscape such as those found in Nebraska, USA. To approximate a landscape spatial scale that is biologically relevant we selected a 5 km radius window, which is roughly equal to the dispersal distance of a Ring-necked Pheasant [49]. We calculated the proportion of each land cover within the specified window size surrounding the survey point (Table 1). Because pheasants likely responded to topographic relief in an area rather than elevation above sea level, we quantified the relative elevation in the surrounding area by deriving an elevation index from a Nebraska digital elevation model (DEM) with a 30×30-m resolution. The elevation index was equal to the standardized elevation of a township, where the average elevation within a congressional township (*j*) is subtracted from each individual raster cell (*i*) and was divided by the standard deviation of elevation within the township [63].
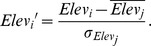



**Table 1:** The range, mean, standard deviation and median values indicating the proportion of a land cover type within a spatial scale relevant to habitat management (1 km radius) and the surround landscape (5 km radius).

Variables	Min	Mean	Stand. Dev.	Median	Max
CRP 1 Km	0.00	0.08	0.09	0.04	0.46
CRP 5 Km	0.00	0.06	0.05	0.06	0.22
Grass 1 Km	0.11	0.48	0.21	0.45	0.99
Grass 5 Km	0.07	0.45	0.16	0.44	0.81
Row crop 1 Km	0.00	0.21	0.19	0.14	0.75
Row crop 5 Km	0.07	0.33	0.19	0.27	0.82
Small grains 1 Km	0.00	0.05	0.08	0.02	0.45
Small grains 5 Km	0.00	0.08	0.07	0.06	0.30
Trees 1 Km	0.00	0.08	0.09	0.04	0.46
Trees 5 Km	0.00	0.06	0.05	0.06	0.22
Wetland 1 Km	0.00	0.03	0.08	0.00	0.38
Wetland 5 Km	0.00	0.01	0.01	0.00	0.05

Land cover and topographic variables were quantified using spatial scales relevant to the managed area and the landscape surrounding the management area. Because there were differences in scale (*i.e.,* the range and composition of values for land cover variables are different from those associated with the topographic variable), all variables were standardized by subtracting the mean and dividing by the standard deviations from the mean [63]. In addition, standardizing variables helps improve model convergence and allowed for the direct comparison of parameter estimates [64]. Before including land cover and topographic variables, we tested all variables for colinearity (Table 2). Any two variables measured within the same spatial scale having a Spearman rank correlation coefficient ±0.6 were determined to be correlated [65] and we eliminated one of the variables based on whether it was correlated with other explanatory variables, was less likely to constrain the scope of potential management response for the species, or was less supported by previous literature.

**Table 2:** Pairwise Spearman’s ranked correlation Rho statistics for land cover variables.

	crp 1-km	crp 5-km	grass 1-km	grass 5-km	row crop 1-km	row crop 5-km	small grains 1-km	small grains 5-km	trees 1-km	trees 5-km	wetland 1-km	wetland 5-km
crp 1-km	1.0											
crp 5-km	0.8	1.0										
grass 1-km	−0.2	0.0	1.0									
grass 5-km	0.0	0.1	0.7	1.0								
row crop 1-km	0.0	−0.2	−0.7	−0.6	1.0							
row crop 5-km	−0.2	−0.4	−0.6	−0.7	0.7	1.0						
small grains 1-km	0.2	−0.1	−0.1	0.2	0.3	0.2	1.0					
small grains 5-km	0.1	−0.2	0.1	0.5	0.1	0.0	0.7	1.0				
trees 1-km	−0.1	0.3	0.2	−0.1	−0.4	−0.3	−0.3	−0.4	1.0			
trees 5-km	0.2	0.6	0.2	0.0	−0.5	−0.5	−0.3	−0.3	0.8	1.0		
wetland 1-km	−0.1	−0.4	−0.5	−0.5	0.5	0.6	0.0	−0.1	−0.3	−0.5	1.0	
wetland 5-km	−0.3	−0.5	−0.3	−0.4	0.4	0.6	−0.1	−0.1	−0.3	−0.4	0.7	1.0

### Statistical Model

**Figure 2:**
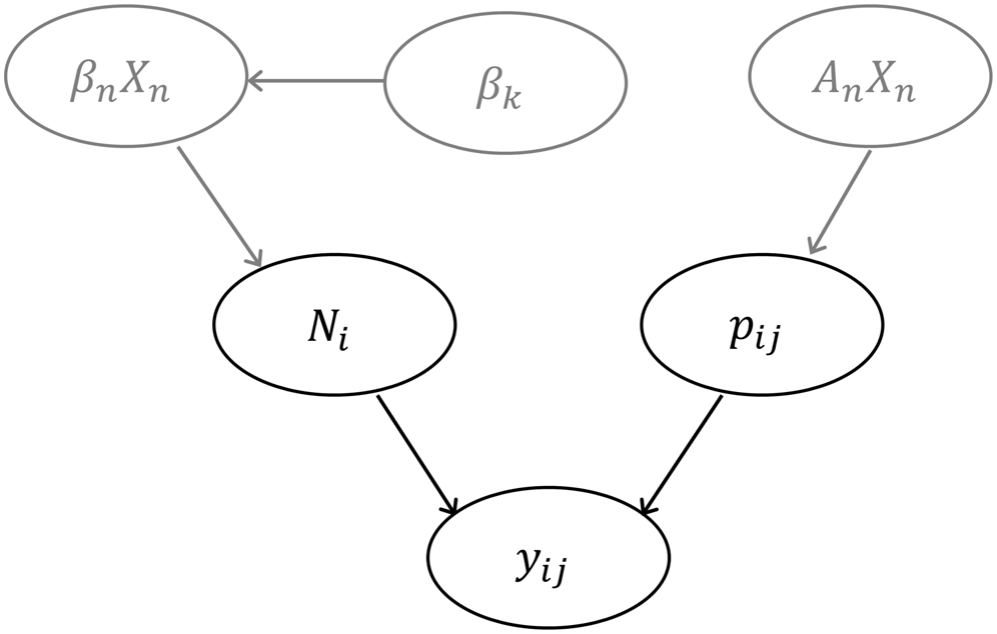
A directed acyclic graph describing the hierarchical Bayesian binomial-Poisson model used to assess the relationships between various land cover variables and Ring-necked Pheasant abundance. Black nodes represent the non-covariate structure and the gray nodes represent the covariate structure. Notation: *y_ij_* is the number of pheasants detected at survey site *i* during the *jth* survey and represents the product of a binomial distribution given the probability of detecting an individual (*p_ij_*) and the number of individuals truly present was *N_i_*. The detection probability, *p_ij_*, at site *i* during the *jth* survey is a logit-linear function of covariates 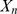 and parameter estimates 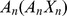. It is assumed that *N_i_* is Poisson distributed with a mean of *λ*. Mean abundance at site i is a function of site-specific covariates 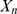 with a random intercept 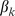 and a slope of 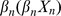.

We modeled relative abundance (*N_i_*) for Ring-necked Pheasant at each survey site (*i*) using a binomial-Poisson hierarchical model which is particularly useful in both predicting species abundance and identifying what habitat and landscape attributes are truly affecting species abundance [64], [66], [67], [68]. By making full use of the repeated visits to each survey point during a survey season, a binomial-Poisson hierarchical mixture model estimates true species abundance corrected for imperfect detection [64], [66], [67], [68]. In addition, by using a Bayesian framework and Markov Chain Monte Carlo (MCMC) simulations, we were able to integrate survey site as a random effect in the model, accounting for the hierarchical structure of the data resulting from the sampling design [69]. The model assumes a two stage stochastic process, where the first stochastic process relates to the ecological processes involved in distributing individuals throughout the landscape resulting in site specific abundance, *N_i_*. We assumed that *N_i_* was Poisson distributed which is an appropriate choice for count data [69] and had a mean of *λ*. We further evaluated the appropriateness of using a Poisson distribution for count data by comparing the sample quantiles to theoretical quantiles from a normal distribution by creating a quantile-quantile plot [69] (Figure S1). We included land cover and topographic variables in the linear predictor for the ecological process using a log-link function for *λ*. Because survey locations were visited repeatedly and nested inside management area *k*, we added a random-intercept effect to account for potential spatial autocorrelation and variation among management areas [69]. We further assessed the effects of spatial autocorrelation on both the raw abundance data (maximum number of individuals detected per three visits) and the residuals by evaluating Moran’s I over multiple distance bands in a correlogram [70] (Figure S2). Moran’s I values range from –1 to 1, with values close to 0 representing a random spatial pattern and values –1 and 1 representing perfect dispersion and perfect correlation, respectively [71]. The second stochastic process in the model is the observation process, where the actual number of individuals detected at site *i* during the *j*th survey (*y_ij_*) was the product of a binomial distribution given that there were *N_i_* individuals present at site *i* and a probability of detecting those individuals *p_ij_* [67] (Figure 2). This model had the general form:
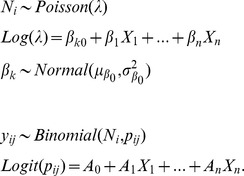



We predicted that survey specific variables, time of day and Julian date, would influence the probability of detecting individuals [57], [72], [73] and therefore included them in the observation process using a logit-link function for *p_ij_*. Peak vocalization-rates have been previously identified [57]; therefore we added a quadratic term for time of day to allow for non-linear relationships in detection probability.

We ran the Bayesian analysis in WinBUGS [73] using the R2WinBUGS package through the software R version 3.0.2 [74]. Three MCMC simulation chains were used to calculate the posterior distribution with 35,000 iterations in each chain. Every 50^th^ iteration was used to calculate the posterior distribution. We treated the first 5,000 iterations of the Markov Chain as a burn-in period and eliminated them from the calculation of the posterior distribution [67]. We visually inspected the Markov Chains and used the Gelman-Rubic diagnostic, which compares within-chain and between-chain variability to determine model convergence [75]. Any parameter estimate with a Gelman-Rubic diagnostic below 1.1 was accepted as having successfully converged.

Model fit was assessed using a posterior predictive check using a Chi-squared discrepancy test [67], [76]. We compared the lack-of-fit of the model fitted with the actual dataset with the lack-of-fit of a model fitted with replicated data generated from the parameter estimates obtained from the actual model. A Bayesian p-value was calculated to further assess model performance, which quantifies the proportion of times the discrepancy measure for the replicated dataset is greater than the discrepancy measure for the actual dataset [67]. For example, a Bayesian p-value near 0.5 would indicate a good performing model.

### Determining Spatial Scale

Land cover variables were measured using two spatial scales relevant to either land management (314 ha), or the landscape (7,854 ha), which was selected using the average between-season dispersal distance of a Ring-necked Pheasant [49]. The percentage of each land cover variable within the surrounding area was quantified using a 1 km and 5 km radius moving window analysis respectively [77].

Previous studies have utilized various information-theoretic approaches (*i.e.,* AIC, BIC, DIC) to identify the spatial scales and cover types important in explaining species occupancy or abundance [18], [78]. Unfortunately, the performance of information-theoretic approaches is controversial when applied to a Bayesian hierarchical modeling [79], [80], [81]. Instead we used a hypothesis testing approach to build a mixed-scaled model, identifying which spatial scale our land cover variables had the strongest influence on Ring-necked Pheasant distribution based on the strength of the parameter estimates [82]. We first modeled all of the variables measured at the management scale (314 ha), created a second model with all of the variables measured at the landscape scale (7854 ha), and assessed which parameter estimates for a single cover type better fit the Ring-necked Pheasant abundance data. Since the majority of land cover variables were highly correlated with themselves across both spatial scales (*i.e.,* grassland at 1 km was highly correlated at 5 km; Table 2), we model the 1 km and 5 km variables separately. Furthermore, we wanted to identify which spatial scale best explained the variability in Ring-necked Pheasant abundance. By separating the two scales we gained a better understanding of how each variable influenced abundance. The spatial scale at which the land cover variable had a stronger relationship and was biologically sensible was included in the final mixed-scaled model (Table 3). Because we were directly comparing parameter estimates to identify an appropriate scale, we did not allow for non-linear land cover relationships during our hypothesis testing approach. However, in the mixed-scale model we added a quadratic term for all land cover variables measured within a 5 km radius of the survey location. We assumed all of the effects within the mixed-scale model were present, circumventing the use of an information-theoretic approach in model selection [76], [80].

**Table 3:** Parameter estimates of habitat and topographic variables measured at the management (1 km radius) and landscape scales (5 km radius), and the mixed-scale model with habitat variables measured at both the management and landscape spatial scales.

			Mixed-scale model				
Variable	1-km scale estimates	5-km scale estimates	Final model estimates	SD	95% credible interval		Final scale (km radius)
					2.5%	97.5%	
intercept	2.98	2.84	3.07	0.60	1.86	4.10	-
CRP	0.44	0.10	0.23	0.08	0.08	0.38	1
grass	0.39	0.22	0.13	0.08	−0.03	0.29	1
wetland	0.21	−0.22	−0.10	0.09	−0.28	0.06	1
trees	−0.11	−0.44	−0.55	0.13	−0.79	−0.27	5
trees^2^	-	-	0.13	0.08	−0.02	0.29	5
row crop	0.46	0.65	0.51	0.18	0.16	0.87	5
row crop^2^	-	-	-0.05	0.09	−0.22	0.15	5
small grains	0.22	0.42	0.45	0.14	0.18	0.72	5
small grains^2^	-	-	−0.04	0.05	−0.14	0.06	5
elevation index	−0.09	−0.03	−0.07	0.05	−0.17	0.04	-
year	−0.09	−0.11	−0.16	0.13	−0.38	0.11	-

### Spatial Modeling and Validation

We created a predictive spatially-explicit model, enabling state-wide predictions of pheasant abundance, by integrating our best statistical model with our independent land cover and topographic variables using a geographic information system (ArcGIS 10.0, Environmental Systems Research, Redlands, CA). Since the statistical models were fit on transformed covariates, the resulting model parameters had to be back-transformed in order to be applied to the covariate data (to predict state-wide abundance) by using the means and standard deviations of each variable in the ArcGIS Spatial Analyst calculator. The resulting weighted raster layers were summed together and added to the intercept, producing a species distribution model for Ring-necked Pheasants in Nebraska [78].

**Figure 3:**
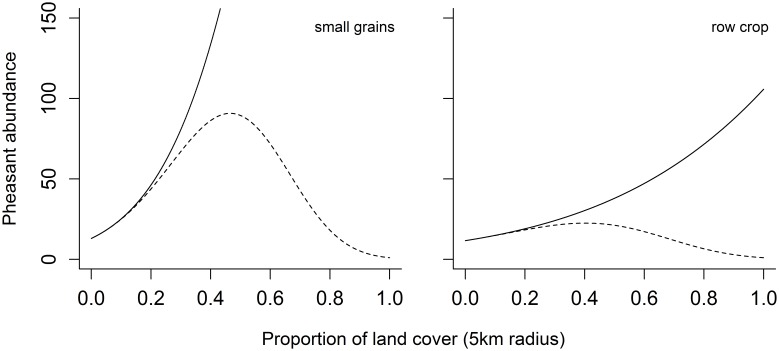
The fitted and corrected relationships between Ring-necked Pheasant abundance and crop types in the surrounding landscape. Fitted relationships for Ring-necked Pheasant counts indicated a positive response to small grains and row crops in the landscape (dark line), but failed to predict pheasant response in areas containing a higher proportion of either cover class located outside of the study region. The range of data values used to fit the relationship between Ring-necked Pheasant abundance and row crop is 0.00–0.75 and a mean of 0.25. The range of data values used to fit relationship between Ring-necked Pheasant abundance and small grains is 0.0–0.45 and a mean of 0.08. Assuming that too much row crop or small grains in the landscape is detrimental to pheasants, dashed lines represent the corrected relationships used to create the final spatial model of Ring-necked Pheasant abundance in Nebraska.

Upon closer examination of the land cover relationships on abundance and inspection of the species distribution model, which was created using the fitted values from the statistical model, we recognized that certain land cover relationships did not make biological sense based on the biology of the species and the ecotypes of the region. Specifically, land cover variables such as row crop and small grains had a strong positive relationship with pheasant abundance; yet previous studies have demonstrated that while both variables benefit pheasants, too much of either land cover leads to a decline [83], [84]. To adjust the species distribution model for Ring-necked Pheasants, we assumed that landscape variables may not adequately identify non-linear relationships (*i.e*., pheasants may benefit from a certain percentage of small grains but not too much), and we added an additional term (cubic term) for small grains and row crop, which was manually added during post statistical modeling efforts. We adjusted the relationship by constraining the model with the assumption that zero Ring-necked Pheasants occur in areas containing 100% small grains or row crop agriculture in the surrounding landscape (Figure 3) [83], [84]. By assuming constant values for all variables in the model and setting row crop to 100%, we added a cubic term for row crop and set *y*, the predicted number of pheasant at a location, equal to 0. We then back-solved for the cubic coefficient, and repeated the procedure for small grains. We used the resulting model as our corrected species distribution model for Ring-necked Pheasants.

We evaluated the spatial models, which predicted pheasant abundance beyond our original sample area, using our independent dataset. Although other validation methods utilize data from the original dataset (e.g., k-fold cross-validation), we used an independent dataset instead, which may more adequately gage model performance [18]. Furthermore, our independent dataset was collected using a slightly different sampling design (roadside surveys) which led us to not include the independent dataset with the rest of the training data used to fit the statistical models but gave us an excellent opportunity to test the generality of our model. We extracted values of both the fitted spatial model and “corrected” spatial model to the survey points of each transect using ArcGIS [85]. We calculated Spearman’s rho statistic for ranked correlation (r_s_) between the observed dataset and the predicted datasets using the statistical software program R [74]. Since the N-mixture model accounts for failing to detect individuals when indeed an individual or multiple individuals were present, the predicted number of birds at a location does not necessarily reflect what was observed. Therefore we felt that using Spearman’s rho statistic (r_s_) to compare relative abundance more adequately assessed model performance. In order to visually inspect model performance, we used standardized observed abundance and standardized predicted abundance to fit a least-squares regression line and 95% confidence limits [85]. The standardized values represent the number of standard deviations from the mean for each dataset. We evaluated both the fitted and the “corrected” spatial model further by calculating the root mean square error (RMSE) for each model [86], [87], [88]. RMSE values are indicative of the sample standard deviation of the differences between the standardized predicted and observed values of Ring-necked Pheasant abundance.

## Results

**Figure 4:**
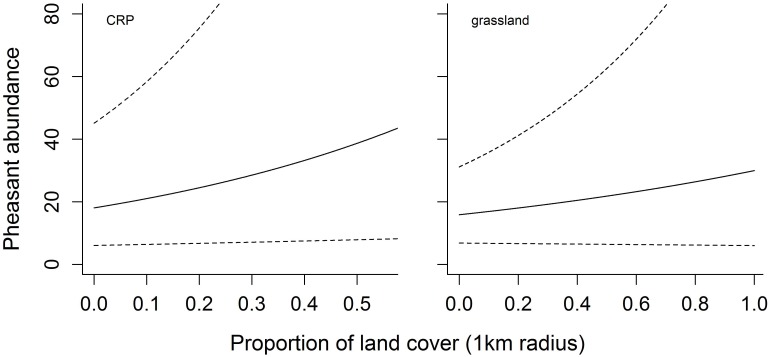
The relationships between Ring-necked Pheasant abundance and the proportion of land cover types within a1 km radius. Ring-necked Pheasant populations respond positively to the proportion of CRP (a) and grassland habitat (b) at the local management level (1 km radius). Solid line represents land cover relationships and the dashed lines represent the 95% credible intervals predicted out to the maximum range we observed during the study.

**Figure 5:**
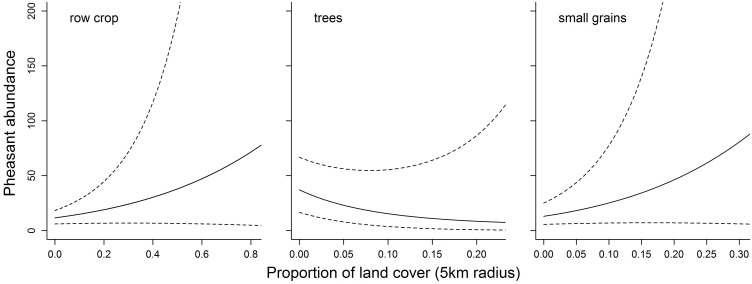
The relationships between Ring-necked Pheasant abundance and the proportion of land cover types within a 5 km radius. Ring-necked Pheasant populations respond positively to the proportion of row crop agriculture and small grains within the landscape (5 km radius), but negatively to the proportion of trees in the landscape. Solid line represents land cover relationships and the dashed lines represent the 95% credible intervals predicted out to the maximum range we observed during the study.

**Figure 6:**
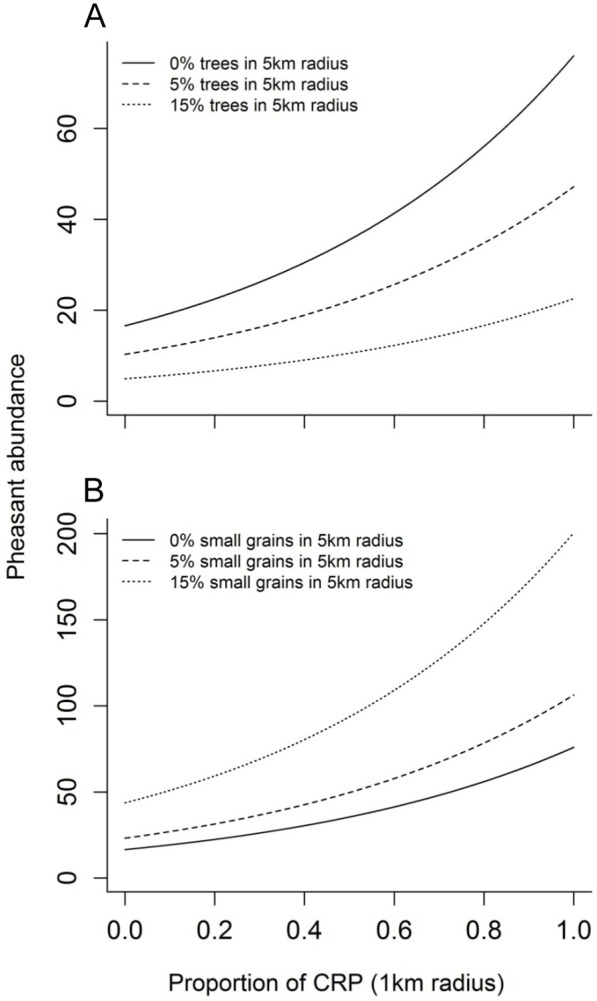
The change in Ring-necked Pheasant response to CRP enrollment as the saturation of trees or small grains varies in the surrounding landscape (5 km radius). CRP enrollment increases pheasant abundance; however the benefits of CRP are inhibited by trees (a) in the surrounding landscape while aided by small grains (b). Solid line represents null relationship of CRP and pheasant counts. Dotted lines represent additive effects of the second cover type in the landscape.

Of the seven topographic and land cover variables we investigated, the proportion of CRP and grass best explained the variability in pheasant abundance at the management scale (Figure 4), with pheasant populations responding positively to each. In contrast, row crop agriculture, small grains and trees best explained the variability in pheasant abundance at the landscape scale (Figure 5), with pheasant populations responding positively to the proportion of row crop and small grains in the landscape, but negatively to the amount of trees such that as few as 15% trees in the landscape severely limited the population (Figure 6). When combined in the mixed-scale model, the landscape-level variables better predicted Ring-necked Pheasant abundance than local-scale variables relevant to management actions (Table 3).

Overall the assessment of model fit for the Bayesian binomial-Poisson mixture model, which included a combination of variables quantified at local and landscape scales, indicated a well preforming model (Bayesian P-value = 0.57). Visual assessment of the chi-squared discrepancy test indicated that the lack-of-fit of the fitted model was comparable to the lack-of-fit of the replicated data generated from the parameter estimates.

**Figure 7:**
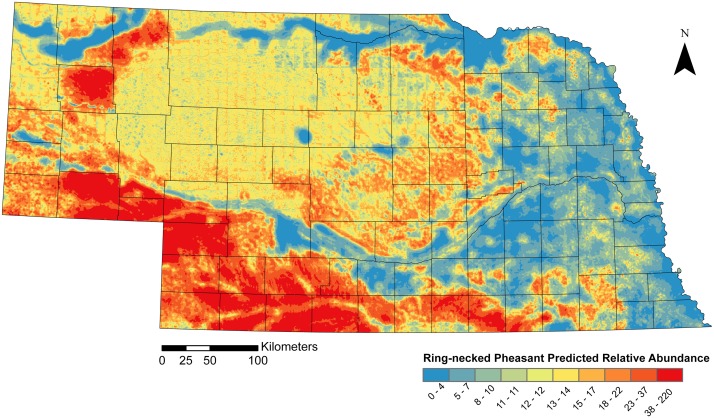
The final 30×30-m resolution predicted Ring-necked Pheasant species distribution model for Nebraska based on the corrected fitted land cover and topographic variables. The range of predicted values was divided into ten categories based on an equal area approach, whereas each color class represents 10% of the area within the entire species distribution model. Classifying the relative predicted abundance values using this approach allows users to pinpoint the top 10% of the areas within the Nebraska that contain the highest predicted abundance (bright red), which is useful in management planning and implementation.

**Figure 8:**
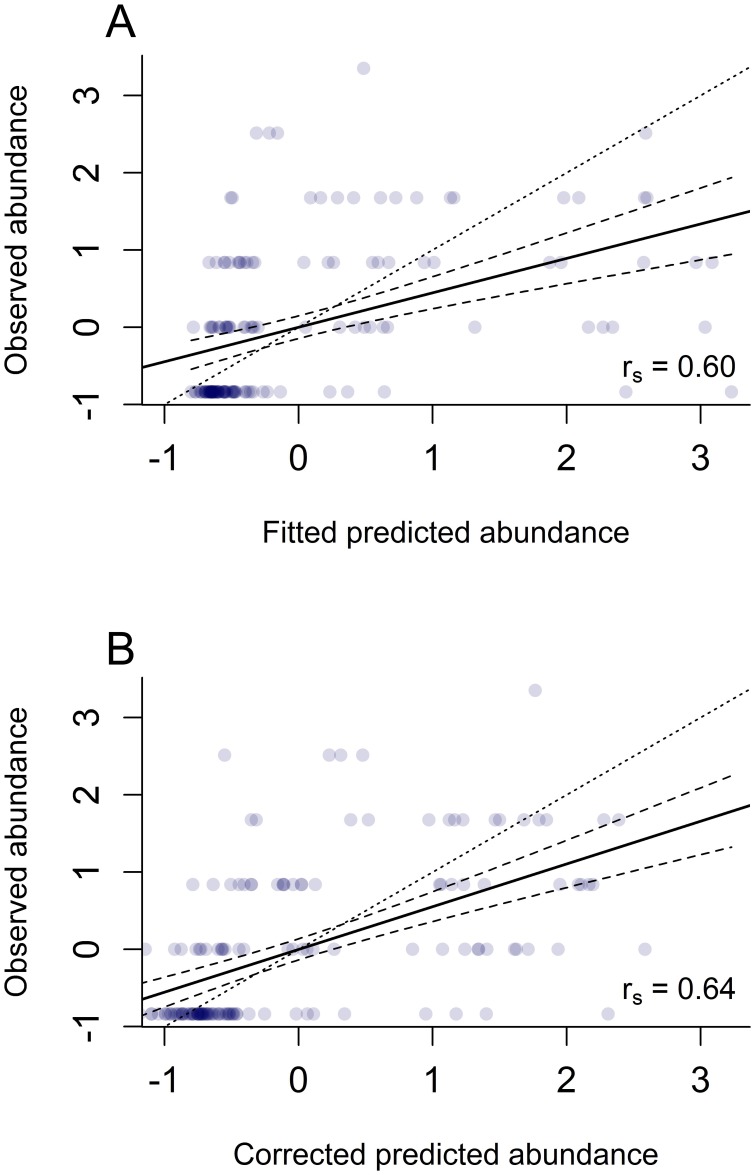
The evaluation of the predictive performance of the Ring-necked Pheasant fitted and the corrected species distribution models. Standardized predicted values of Ring-necked Pheasant abundance compared to observed abundance values from an independent dataset collected in 2012 indicated that both the original spatial model (A) and the corrected spatial model (B) perform well. Data points are identified in blue, where the intensity of points is reflected by the color shade (dark blue = high intensity, and light blue = low intensity). The solid black line represents the fitted least-squares regression line and the two dashed lines represent the 95% confidence intervals. The dotted line identifies where a perfect fit would occur between predicted pheasant abundance and observed abundance.

Based on the corrected species distribution model, Ring-necked Pheasant populations were predicted to be most abundant in the southern and southwestern regions of Nebraska (Figure 7). Concentrations of abundance also occurred around Alliance, Nebraska, located in the panhandle region of the state. Spearman’s rho correlation statistics for the SDM based on the fitted model (r_s_ = 0.60) and the SDM based on the corrected land cover relationships (r_s_ = 0.64) indicated that both models predicted pheasant abundance across Nebraska, particularly at lower abundances, including outside the primary study area (Figure 8) [85]. The RMSE for the SDM containing the corrected land cover relationships (RMSE = 0.94) was also less than the fitted model (RMSE = 1.05). Because of its higher r_s_ statistic and its lower RMSE, we identified the SDM constructed from the corrected land cover relationships as being the better model. In addition, the range of inputs used to derive our fitted model did not match the range of land cover values throughout the state.

Regions within the SDM containing drastic elevation differences, such as the strong topographic relief found in Nebraska’s Sandhills region (North-central region of the state), have led to an uneven prediction gradient, or stripping effect, for predicted abundance values within the SDM (Figure 7). This phenomenon is an artifact of calculating the elevation index by taking the difference from local elevation in a DEM and the mean within a township and dividing by the standard deviation. The majority of the differences between the predicted values associated with this stripping effect amount to only a few individuals (Figure 7).

## Discussion

The influence of local habitat conditions, and thus habitat management on population viability and productivity is clear [25], [42], [61], [89], [90]. However, while local conditions are obviously important, species are likely to respond to ecologically relevant conditions across multiple spatial scales [30], [91], [92]. For Ring-necked Pheasant, not only did we find that populations were responding to unique ecological conditions at different spatial scales, we clearly demonstrate the capability of large scale conditions to both facilitate and constrain local habitat benefits. For example, it is not surprising that the availability of grassland habitats at the local level had a positive influence on pheasant abundance (Figure 4), but the strength of these land cover relationships were significantly constrained by relationships at the landscape scale (Figure 6). Several studies have previously suggested that local habitat management is critical for pheasant populations [24], [90] – the “if you build it, they will come” approach – but our findings show the benefits of these actions are constrained by presence of trees in the landscape and facilitated by the availability of row crop and small grains, at least to a point (Figure 6). Based on these results, we suggest the interspersion of local grassland patches within landscapes containing small grains and even row crop agriculture is a critical element in maintaining Ring-necked Pheasant populations.

The presence of small grains, for example, is widely known to aid breeding success of Ring-necked Pheasants [52], [93], often accounting for a significant proportion of productivity even when limited in availability in the landscape [94]. In agriculturally dominated landscapes where nesting habitat is significantly limited, the early green-up and ‘grass-like’ habitat created by small grains such as winter wheat may significantly increase breeding opportunities, a major factor limiting pheasant populations [94], [95]. Small grains may be beneficial as nesting cover (Figure 6), but they have limited benefits for brood rearing because arthropod food resources are generally reduced by agriculture practices [96]. And, the winter cover afforded by grain stubble is significantly less than native warm season grasses [44], [98]. Similar trade-offs are apparent for row crop habitats which produce ideal winter food resources [98], [99], but have limited benefits as breeding or winter cover [84], [97].

The inability of small grain and row crop cover classes to fulfill all the life history requirements of pheasants underlies our assumption that at some point the benefits associated with increasing dominance of agriculture in the landscape are offset by the costs, creating a normal distribution around some ideal availability of small grain and row crop. Based on the fitted relationships for row crop and small grain habitat types, the initial Ring-necked Pheasant SDM was inflated in areas where extremely high proportions of these cover types existed in the landscape. This “run-away” regression error was an artifact of extrapolating beyond the study region, where elevated cover class values were not used in fitting the statistical model (Figure 3). It is acknowledged that modeling the spatial distribution and abundance of species is largely an *ad hoc* process [78] and by introducing habitat relationships based on the biology of the species, we were able to correct the fitted relationships for landscape variables and improve the performance of the SDM (Figure 8). This approach bridges the gap between habitat suitability indices and regression-based species distribution modeling, in that habitat suitability indices are largely based on *a priori* knowledge of the species of interest and expert opinion [18]. It is widely held that probabilistic modeling is required to adequately model species distributions [100]; yet, we have demonstrated that by combining both a conceptual and empirical approach to species distribution modeling, we can reasonably predict species abundance and distribution based on known ecological trade-offs. Moreover, these trade-offs highlight the cross-scale interactions apparent in our model and demonstrate the importance of ecological processes which act across spatial scales.

An example of an ecological process that works across spatial scales and which may be highlighted by the findings of our model is nest predation [101]. Nest predation is the primary cause of reproductive failure for most birds [102], [103] and, thus, represents an important factor limiting pheasant populations. In the grassland ecosystems of Nebraska the primary nest predators limiting pheasant nest success are mesopredators (e.g., raccoon, skunk, possum) [104], [105], [106], most of which are limited by the availability of adequate winter and breeding habitats afforded by large trees [107], [108]. Thus while other studies have suggested that mature woody cover benefits pheasants [50], we found that even limited woody cover in the landscape has strong negative consequences to pheasant populations (Figure 5). This finding is likely driven by anthropogenic impacts to the landscape that alter predator-prey interactions, particularly predator search strategies. In highly altered and intensively managed agroecosystems nesting cover is generally limited, allowing highly mobile nest predators to converge and concentrate search effort [109]. Thus even small increases in nest predator populations, mediated by small increases in woody cover, have detrimental and lasting impacts on pheasant populations. Improving nest success requires reducing nest predator populations [110], [111], potentially by removing trees, or reducing nest predator efficacy [112]. Indeed, the latter possibility likely underlies the positive impact of small grains in the landscape, which increase predator search area and likely nest dispersion, both of which reduce the positive feedback-loop inherent in predator search effort [113]. Clearly, the complex factors driving nest success and consequently pheasant abundance are mediated by multiple ecological factors working across multiple scales.

The rate of decline in populations of grassland and farmland birds is alarming [39], [40]; however despite increasing conservation efforts over the last thirty years, particularly local habitat management [38], [41], [43], most populations continue to decline. As conservation efforts are sometimes perceived as failures [12], [14], [44], and sources of funding become more limited and increasingly coveted for alternative needs [114], [115], [116], [117], a loss of public support may underlie a reduction in future conservation efforts [35], [36]. To improve management efficacy and ensure the long-term sustainability of conservation, biologists must identify the ecological factors that constrain management success. The importance of the landscape-level effects suggests that local-scale land management is not likely the driving factor influencing pheasant populations. It is important to note, however, even though our two spatial scales were seemingly different and were based on the biology of the species, and typical land management actions, the land cover variables were highly correlated with their complement across spatial scales (Table 2). The high collinearity between the two land cover variables (i.e., percent grass measured within 1-km radius and percent grass measured at 5-km radius) makes it challenging to say for certain which spatial scale it driving pheasant abundance. Still, the reasonably adequate performance of the pheasant SDM supports our conclusion, as we were able to predict a completely independent dataset of observed pheasant numbers based on a model fitted from data collected only on managed lands (Figure 8). By identifying and understanding how species select habitat and at what scales, we were better able to predict species distribution and pinpoint how populations may respond to management decisions on a local level. Although many species may respond to habitat characteristics at spatial scales too small to identify using GIS technology, here we demonstrated the importance of identifying spatial relationships to better understand and predict species distribution and ultimately improve the management outcome for species responding to habitat beyond the boundaries of a management area.

These findings contribute to our ability to effectively manage for Ring-necked Pheasant populations in Nebraska by increasing our understanding of how populations respond to management efforts. Our results show that pheasants responded positively to local habitat management such as CRP enrollment (Figure 4). However, the landscape context surrounding management areas had drastic ramifications on the outcomes of local management efforts (Figure 6). For instance, our findings demonstrate that areas in the landscape containing a high proportion of trees may in fact inhibit any benefits of local management efforts on Ring-necked Pheasants. Alternatively, managing habitat in areas suitable for Ring-necked Pheasant populations, such as in landscapes containing a high proportion of small grains, will enhance the benefits of local management (Figure 6).

Our results support current efforts to manage at the landscape scale, when possible [118]. On private lands, groups of land owners may be encouraged to cooperate and form “conservancies” to coordinate efforts at the landscape-scale. Agencies may also provide incentives to private lands in selected watersheds, areas of conservation concern, or “hot spots” to create effective management outcomes. And, Public land managers can use SDMs to select lands for acquisition by pin-pointing, visually, areas in the landscape that have the highest likelihood of a successful outcome given a management action [118]. Public managers can apply this theory to small parcels of public land by creating relationships with neighboring landowners and funneling incentives for conservation to these landscapes, thus potentially improving their success rate at maintaining and increasing populations [118] (Figure 7). As conservation resources become increasingly limited, targeted, prescribed management at the landscape level is necessary to get the most bang for the conservation dollar.

## Supporting Information

Figure S1 A quantile-quantile plot comparing the residuals from the binomial-Poisson hierarchical model to a normal distribution. The residuals from the binomial-Poisson hierarchical model used in modeling Ring-necked Pheasant abundance match up closely to quantiles from a theoretical normal distribution (solid black line). The close relationship between the sample and theoretical quantiles indicates that a Poisson distribution was an appropriate distribution for modeling Ring-necked Pheasant abundance.
(DOCX)

Figure S2 A correlogram quantifying the amount of spatial autocorrelation at varying distances between survey locations using raw abundance data for Ring-necked Pheasants and the residuals from the binomial-Poisson hierarchical model. The effects of spatial autocorrelation (both negative and positive) is visually apparent for the raw abundance data (red line) for Ring-necked Pheasant by inspecting the correlogram, which calculated Moran’s I for every 2,500 m interval out to 500,000 m. Moran’s I values range from −1 to 1, with values close to 0 representing a random spatial pattern and values −1 and 1 representing perfect dispersion and perfect correlation, respectively. The maximum abundance was calculated as the maximum number of Ring-necked Pheasants detected at a survey location after three repeated visits (red line). The residuals from the binomial-Poisson hierarchical model (blue line) indicate that all of the spatial autocorrelation was effectively accounted for by including survey site as a random variable in the model.
(DOCX)

List S1 A list of GPS coordinates for each Ring-necked Pheasant survey site across southern Nebraska.
(DOCX)
