# Supplementary figures and images for: Assessing Landscape Constraints on Species Abundance: Does the Neighborhood Limit Species Response to Local Habitat Conservation Programs?
Source: PLoS One. 2014 Jun 11;9(6):e99339. doi: 10.1371/journal.pone.0099339 (PMC4053377; doi:10.1371/journal.pone.0099339)

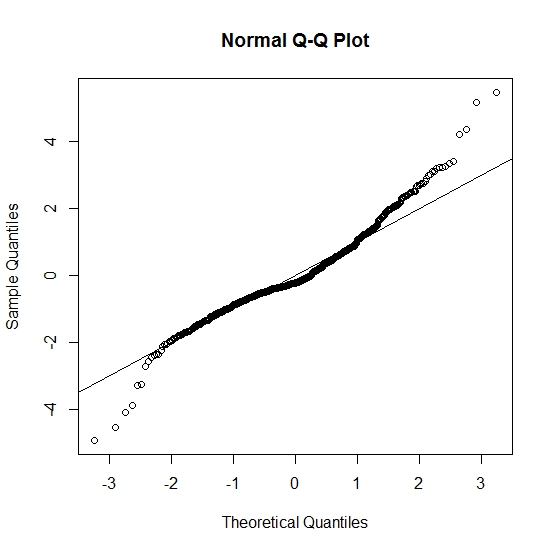

Supplement: Figure S1 — A quantile-quantile plot comparing the residuals from the binomial-Poisson hierarchical model to a normal distribution. The residuals from the binomial-Poisson hierarchical model used in modeling Ring-necked Pheasant abundance match up closely to quantiles from a theoretical normal distribution (solid black line). The close relationship between the sample and theoretical quantiles indicates that a Poisson distribution was an appropriate distribution for modeling Ring-necked Pheasant abundance. (DOCX) [file pone.0099339.s001.docx]

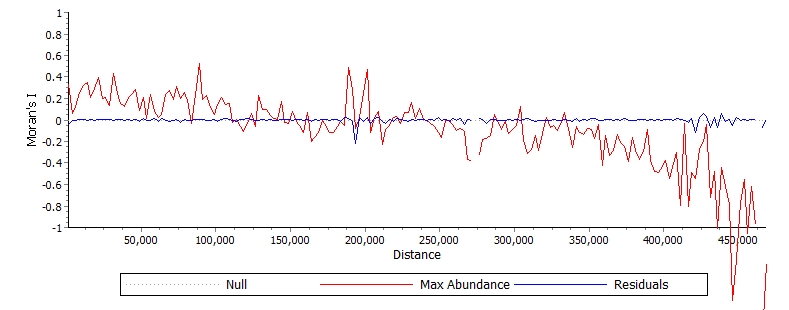

Supplement: Figure S2 — A correlogram quantifying the amount of spatial autocorrelation at varying distances between survey locations using raw abundance data for Ring-necked Pheasants and the residuals from the binomial-Poisson hierarchical model. The effects of spatial autocorrelation (both negative and positive) is visually apparent for the raw abundance data (red line) for Ring-necked Pheasant by inspecting the correlogram, which calculated Moran’s I for every 2,500 m interval out to 500,000 m. Moran’s I values range from −1 to 1, with values close to 0 representing a random spatial pattern and values −1 and 1 representing perfect dispersion and perfect correlation, respectively. The maximum abundance was calculated as the maximum number of Ring-necked Pheasants detected at a survey location after three repeated visits (red line). The residuals from the binomial-Poisson hierarchical model (blue line) indicate that all of the spatial autocorrelation was effectively accounted for by including survey site as a random variable in the model. (DOCX) [file pone.0099339.s002.docx]
