# Supplementary material for: Assessing Landscape Constraints on Species Abundance: Does the Neighborhood Limit Species Response to Local Habitat Conservation Programs?
Source: PLoS One. 2014 Jun 11;9(6):e99339. doi: 10.1371/journal.pone.0099339 (PMC4053377; doi:10.1371/journal.pone.0099339)
Supplement: List S1 — A list of GPS coordinates for each Ring-necked Pheasant survey site across southern Nebraska. (DOCX) [file pone.0099339.s003.docx]

GPS coordinates projected in North American Datum 1983 zone 14: E 640963, N 4455090; E 722781.9, N 4439717; E 718762.4, N 4438698; E 687307.8, N 4500071; E 714420.6, N 4461076; E 356355, N 4509980; E 342819.7, N 4498142; E 681729, N 4434580; E 708101.7, N 4454263; E 646883.1, N 4438025; E 344075.4, N 4468844; E 704537.6, N 4434252; E 285214.7, N 4441397; E 722920, N 4465110; E 334920.1, N 4455916; E 351800.2, N 4447499; E 576991, N 4478000; E 324422.4, N 4464685; E 346702.5, N 4494962; E 739900.9, N 4438038; E 394469 N 4478550; E 634801, N 4452100; E 344162.3, N 4448974; E 344610.8, N 4457453; E 735223, N 4479540; E 727615, N 4435030; E 588970, N 4516100; E 716918.5, N 4471280; E 651325.7, N 4437903; E 725190.9, N 4465247; E 358541, N 4472560; E 478916, N 4469700; E 681279.5, N 4506515; E 744153, N 4467070; E 274445, N 4491390; E 578254, N 4489260; E 473266, N 4467670; E 687203, N 4511660
